# Outcomes of Primary Anterior Cruciate Ligament (ACL) Repair for Proximal Tears: A Systematic Review and Meta-Analysis

**DOI:** 10.7759/cureus.59124

**Published:** 2024-04-27

**Authors:** Collin Braithwaite, Tanner J Hafen, Robert Dean, Amir Lebaschi, Joseph Guettler, James Bicos

**Affiliations:** 1 Department of Orthopedics, Oakland University William Beaumont School of Medicine, Rochester, USA; 2 Department of Orthopedics, Corewell Health William Beaumont University Hospital, Royal Oak, USA

**Keywords:** arthroscopy, acl tear, primary repair, reconstruction, acl

## Abstract

The purpose of this study is to compare failure rates among different techniques of primary anterior cruciate ligament (ACL) repair for the treatment of proximal ACL ruptures. Meta-analysis and systematic review were completed, and Preferred Reporting Items for Systematic Reviews and Meta-Analyses (PRISMA) guidelines were followed. Studies from Embase, Cochrane, and PubMed published between June 2011 and June 2022 reporting outcomes of primary ACL repair on proximal tears with a minimum two-year follow-up were included. Primary ACL repair was divided into dynamic, static, and non-augmented repair. The primary outcome was failure rates, and the secondary outcomes included patient-reported outcomes (PROs) and anterior tibial stability (ATT). Eighteen studies on primary ACL repair were included, with a total of 614 patients (ages ranging from 6 to 65, 60% male). Only two studies were level 1 randomized controlled clinical trials. The static repair had a failure rate of 33 out of 261 (12.6%), non-augmented was 17 out of 179 (9.4%), and dynamic repair was 31 out of 174 (17.8%); no statistically significant difference was found comparing the failure rates (p = 0.090). PROs using the International Knee Documentation Committee (IKDC) and Lysholm scores had weighted averages of 91.7 (95% confidence interval (CI): 89.6-93.8) and 94.7 (95% CI: 92.7-96.7), respectively. ATT had a weighted average of 1.668 mm (95% CI: 1.002-2.334). The primary findings of this paper include a 12.6% combined failure rate for primary proximal ACL repair with no significant difference in failure rate or PROs when accounting for the methodology of repair at a minimum two-year follow-up. It is important to note the lack of high-quality randomized controlled trials, the heterogeneity of included studies, and the lack of long-term data. Despite these limitations, the findings of the current analysis suggest that primary repair may be a useful treatment option for indicated candidates with proximal ACL ruptures. Further long-term and higher-quality comparative studies on ACL reconstruction are warranted.

## Introduction and background

Anterior cruciate ligament (ACL) injuries account for 25-50% of ligamentous sport knee injuries [1]. These injuries pose unique clinical challenges because of their poor capacity to undergo biological healing due to the local intra-articular conditions [2].

In the 1970s, Feagin and Curl presented the outcomes of open primary repair on 64 cadets at the United States Military Academy and reported good to excellent outcomes after two years [3]. Several years later in 1976, however, they reported disappointing mid-term outcomes of the same cohort indicating pain in 71%, swelling in 66%, stiffness in 71%, and instability in 94% of the cohort [4]. In addition, the trend of good short-term outcomes after primary repair with disappointing long-term outcomes was reaffirmed by additional research [5-7]. In the 1980s and into 2000, the focus shifted from ACL repair to ACL reconstruction due to evidence of the latter, resulting in encouraging long-term outcomes [7-10]. Subsequently, several systematic reviews have confirmed the reproducible and reliable long-term outcomes of ACL reconstruction. As a result, primary repair fell out of favor and reconstruction became and continues to be the standard of care for ACL tears [11-13].

However, for a variety of reasons, including the fact that it is less invasive, it is associated with less morbidity, and the theoretically improved proprioceptive potential, there has been renewed interest placed on ACL repair [14,15]. In a 2017 systematic review on open ACL repair, it was noted that the studies with the best outcomes had a larger percentage of proximal tears in their cohorts and that exceptional results were recorded in studies in which the primary repair was only done on proximal tears [16]. The recent resurgence in primary ACL repair research has the potential to challenge the current standard of care for select patients with proximally torn ligaments. We have identified three different methods of primary repair: non-suture augmented repair (NA), static (suture) augmented repair (SA), and dynamic intraligamentary stabilization (DIS) [11]. The purpose of this study was to compare outcomes of different techniques of primary ACL repair in proximal ACL ruptures.

Methods

A systematic review was performed using the Preferred Reported Items for Systematic Reviews and Meta-analyses (PRISMA) guidelines on ACL repair. The query was performed in October 2022. The specific search terms used were “(Anterior Cruciate Ligament OR ACL) AND (repair OR healing OR suture OR reinsertion OR reattachment).”

Eligibility Criteria

The authors searched Embase, Cochrane, and PubMed databases for studies that reported outcomes encompassing the objective and subjective physical exam scores and failure rates for primary ACL repair. Inclusion criteria consisted of (I) outcomes of primary repair; (II) at the minimum, studies had to have a mean or median two-year follow-up; (III) a manuscript published in the English language; (IV) >50% of included participants being treated for a proximal ACL tear; and (V) studies that were published from June 2011 to 2022 (prior systematic reviews have shown no new studies with modern primary ACL repair technique being published before 2014) [16,17]. Exclusion criteria consisted of (I) pre-clinical studies, (II) failure to specify the specific tear location, (III) multiligamentous tears or tibial avulsion fractures, and (V) case reports.

Data Analysis

**Figure 1:**
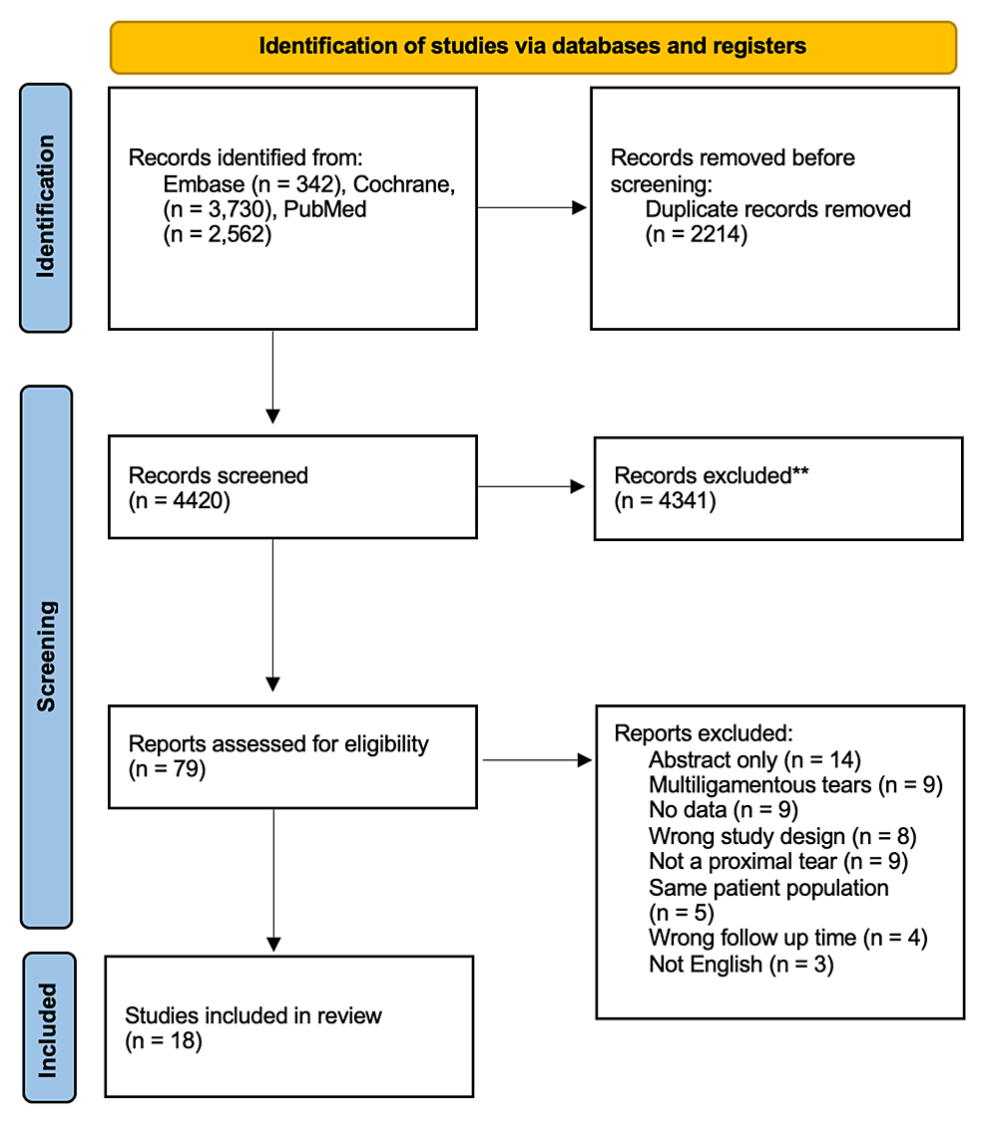
Preferred Reporting Items for Systematic Reviews and Meta-Analyses (PRISMA) flow diagram

The inclusion and exclusion criteria were applied to each article by two independent reviewers (CB, TH). Any disagreements were resolved through a discussion by the two separate reviewers and a third independent reviewer (RD). A summary of this review process is included in Figure 1. The variables extracted were descriptive article information, patient demographics, minimum follow-up time, study population size, ACL tear location, surgical technique, patient-reported outcomes (PROs), number of failures, study definitions of failure, surgical/PROs, anterior-posterior (AP) laxity measurement modalities, and postoperative laxity measurements in millimeters. Studies were included only if they reported on the number of failures. The PROs that were collected were the Lysholm score and International Knee Documentation Committee (IKDC) score. In addition, the repair techniques were grouped into one of three categories: SA, NA, and DIS.

Risk of Bias

All studies were reviewed for bias using the Methodological Index for Non-Randomized Studies (MINORS) tool, which has been previously described and validated [18] (Table 1). The MINORS tool utilized 12 questions to assess the quality of a study, four of which are applicable only to those studies that are comparative. As such, the MINORs tool only utilized eight questions for non-comparative studies. Each of the 12 items was scored 0 to 2: 0, not reported; 1, reported but described or performed poorly or inadequately; 2, reported accurately and well described. Scores of at least 75% were considered high quality with low risk for bias; scores between 50% and 75% were considered medium risk for bias; scores of less than or equal to 50% were considered high risk for bias. For noncomparative studies, the maximum score was 16, while the maximum score for comparative studies was 24.

**Table 1:** Quality assessment using the Methodological Index for Non-Randomized Studies (MINORS) [18]: study that validated the MINORS criteria for assessing bias

Author (year)	Clearly stated aim	Consecutive patients	Prospective data collection	End points appropriate to the aim of the study	Unbiased assessment of end points	Follow-up appropriate to the aim	<5% lost to follow-up	Prospective calculation of the study size	Adequate control group	Contemporary groups	Baseline equivalence of groups	Adequate statistical analysis	Total
Achtnich et al., 2016 [19]	2	2	2	2	0	2	2	2	2	2	0	2	20
Ahmad et al., 2020 [20]	2	2	2	2	0	2	1	1	N/A	N/A	N/A	N/A	12
Bigoni et al., 2017 [21]	2	2	2	2	0	2	2	0	N/A	N/A	N/A	N/A	12
Burton et al., 2021 [22]	2	2	2	2	0	2	1	0	N/A	N/A	N/A	N/A	11
Dabis et al., 2020 [23]	2	2	2	2	0	2	0	2	N/A	N/A	N/A	N/A	12
Douoguih et al., 2020 [24]	2	2	2	2	1	2	2	2	N/A	N/A	N/A	N/A	15
Gagliardi et al., 2019 [25]	2	2	2	2	0	2	1	0	2	2	1	2	18
Heusdens et al., 2019 [26]	2	2	2	2	0	2	1	2	N/A	N/A	N/A	N/A	13
Hopper et al., 2022 [27]	2	2	2	2	1	2	1	2	N/A	N/A	N/A	N/A	14
Hoogeslag et al., 2019 [28]	2	2	2	2	0	2	1	2	2	2	2	2	21
Jonkergouw et al., 2019 [29]	2	2	2	2	0	2	2	0	N/A	N/A			
Kohl et al., 2016 [30]	2	2	2	2	0	2	2	0	N/A	N/A	N/A	N/A	12
Kösters et al., 2020 [31]	2	2	2	2	1	2	2	2	2	2	2	2	23
Liao et al., 2020 [32]	2	2	2	2	0	2	0	0	N/A	N/A	N/A	N/A	10
Liao et al. 2020 [33]	2	2	1	2	0	2	0	0	N/A	N/A	N/A	N/A	9
Mukhopadhyay et al. 2018 [34]	2	2	2	1	0	2	2	0	N/A	N/A	N/A	N/A	11
Turati et al. 2021 [35]	2	1	2	2	0	2	0	1	N/A	N/A	N/A	N/A	10
Vermeijden et al. 2021 [36]	2	2	2	2	0	2	2	0	N/A	N/A	N/A	N/A	14

Statistical Analysis

Continuous variables were extracted and pooled PROs of interest and related standard error were calculated using DerSimonian and Laird random-effect models [37]. Heterogeneity between studies was quantified using the I2 statistic; an I2 statistic value of greater than 75% was used to indicate high heterogeneity. Random-effect models were used for all the analyses. The difference in PRO scores was conducted using the Altman interaction test [38]. Studies were not included in the meta-analysis if they failed to include measurements of variance, and the authors were unable to be contacted to provide these measurements (standard deviation or confidence intervals). In addition, only outcome scores that were reported in ≥3 studies were included in the final meta-analysis. For inclusion in the meta-analysis of ATT measurements, only those that utilized KT-1000 arthrometer measurements were included in the final analysis to help homogenize the data; moreover, those that failed to report measures of variance were not included in this analysis. Failure rates were calculated by adding the number of failures divided by the total number of patients in each cohort. All statistical analyses were conducted using the open-source R software, version 4.0.5 (R Core Team, R Foundation for Statistical Computing, Vienna, Austria). Forest plots were created using OpenMetaAnalyst (Brown University) using the R console package. Dichotomous variables were extracted as the absolute number and percentage, pooled by using the Mantel-Haenszel method [39], and presented as the risk difference (RD) and risk ratio (RR) with a 95% CI.

## Review

Results

Study Identification

The initial literature search resulted in 6,634 studies. A total of 2,214 were duplicates, which left 4,420 unique studies to review. On the title/abstract review, 79 studies were included for full-text review. After the full-text review, 18 studies with a total of 614 patients met the inclusion and exclusion criteria (Figure 1).

Study Demographic Data

A total of 614 patients were included with a minimum two-year follow-up and range of two to 12 years. There were 364 males and 216 females with ages ranging from six to 65 years (two studies did not report gender: Achtnich et al. [19] and Mukhopadhyay et al. [34]) (Table 2).

**Table 2:** Demographic data divided by the type of primary repair Jonkergouw et al. [29] and Vermeijden et al. [36] are listed twice as they both compared SA vs. NA. *Level of evidence is based upon the Oxford classification. **Follow-up reported as the mean or median. ***N/A: relevant data were not included in the paper. ****24-144 months for ACL failure and 24 months for patient-reported outcomes.

Author (year)	Type of primary repair	Level of evidence*	Number of patients	Follow-up** (range in months)	Age range (years)	Average age (years)	% Male	% Proximal tear location
Achtnich et al., 2016 [19]	Non-augmented	3	21	2.3 years (24-31)	N/A***	30	N/A***	100%
Bigoni et al., (2017) [21]	Non-augmented	4	5	3.6 years (25-56)	8-10	9.2	80%	100%
Jonkergouw et al., 2019 [29]	Non-augmented	3	29	3.2 years (N/A)	14-57	37	62%	100%
Liao et al., 2020 [32]	Non-augmented	4	21	3 years (25-49)	17-56	32	76%	100%
Liao et al., 2020 [33]	Non-augmented	4	18	3.1 years (24-52)	18-23	20	83%	100%
Mukhopadhyay et al., 2018 [34]	Non-augmented	4	13	2.6 years (26-38)	21-40	31.3	N/A***	100%
Turati et al., 2021 [35]	Non-augmented	4	19	5.7 years (24-144)****	5-15	9.2	58%	100%
Vermeijden et al., 2021 [36]	Non-augmented	3	53	2.2 years (N/A)	N/A***	35	55%	100%
Burton et al., 2021 [22]	Suture augmented	4	29	2.9 years (24-54)	19-47	32.2	72%	100%
Dabis et al., 2020 [23]	Suture augmented	4	20	2.7 years (29-52)	6-16	12.9	40%	100%
Douoguih et al., 2020 [24]	Suture augmented	4	27	2.8 years (24-46)	N/A***	27.4	67%	100%
Gagliardi et al., 2019 [25]	Suture augmented	3	22	3.2 years (N/A)	7-18	13.9	55%	100%
Heusdens et al., 2019 [26]	Suture augmented	4	42	2 years (24)	14-60	33	57%	100%
Hopper et al., 2022 [27]	Suture augmented	4	34	5.7 years (60-89)	13-60	37.8	53%	100%
Jonkergouw et al., 2019 [29]	Suture augmented	3	27	3.2 years (N/A)	14-57	29.6	55%	100%
Vermeijden et al., 2021 [36]	Suture augmented	3	60	2.2 years (N/A)	N/A**	35	55%	100%
Ahmad et al., 2020 [20]	Dynamic stabilization	4	57	6.3 years (60-90)	20-65	38.6	51%	100%
Hoogeslag et al., 2019 [28]	Dynamic stabilization	1	24	2 years (24)	10-27	21	79%	83%
Kohl et al., 2016 [30]	Dynamic stabilization	4	50	2 years (24)	18-50	30	68%	80%
Kösters et al., 2020 [31]	Dynamic stabilization	1	43	2 years (24)	18-46	28	58%	90%

A total of 261 patients from eight studies received proximal ACL repair by the SA technique [22-27,29,36]. Of the included studies, two compared SA versus NA repair [29,36], and one looked at SA versus reconstruction [25]. Six studies used a transosseous approach with an internal brace [22-27], and one study used two suture anchors drilled into the femoral cortex [36]. Two studies specifically looked at pediatric patients [23,25]. One hundred percent of the patients had a proximal ACL tear.

One hundred seventy-nine patients from eight studies underwent a proximal ACL repair with an NA technique [19,21,29,32-36]. One study compared NA primary ACL repair versus semitendinosus ACL reconstruction [19]. One study specifically looked at pediatric patients [21]. Five studies used one suture anchor to reattach the ACL back to the femoral footprint [19,21,29,35,36]. Two studies used two suture anchors that were placed in the femoral footprint [32,33]. One study used a transosseous approach through the femur and used a cortical button fixed on the lateral femoral cortex [34]. One hundred percent of the patients had a proximal ACL tear.

A total of 174 patients from four studies [20,28,30,31] underwent a proximal ACL repair with DIS. Two studies [28,31] compared DIS ACL repair versus semitendinosus ACL reconstruction and were level I randomized controlled clinical trials. All four studies used the Ligamys intraligamentary stabilization insert, which includes a cortical fixation button on the lateral aspect of the femoral cortex, a polyethylene braid, and a spring-loaded sleeve into the tibial tunnel [20,28,30,31]. Eighty-four percent of the patients had a proximal ACL tear, with the remaining 16% being mid-substance tears.

Study Outcomes

Among 261 patients who underwent proximal ACL repair with SA, the failure rate at the latest available follow-up, and the range (2-7.5 years) was 12.6% [22-27,29,36]. Hopper et al. reported that the six ACL failures were associated with a lower mean age of 20.7 years, which was significantly lower than the average cohort age of 37.8 [27].

Among the 179 patients who underwent proximal ACL repair with the NA technique, the failure rate was 9.4% at the latest available follow-up (range of 2-12 years) [19,21,29,32-36].

There were 174 patients (from four studies) who underwent ACL repair with the DIS technique with a failure rate of 17.8% at the latest available follow-up (range of 2-7.5 years) [20,28,30,31]. Three of the studies had a follow-up time of 24 months [28,30,31], while the last study had an average follow-up of 76 months and a failure rate of 30% [20]. Ahmad et al. [20] also had a higher mean age of 39 years, while the other three were at 28, 30, and 21 [31,30,28].

Failure was defined strictly as requiring revision in two studies [22,24]. The remaining studies considered symptomatic instability or objective laxity as ACL failures in addition to revision ACL reconstruction surgery. One study considered MRI-identified ACL rupture in addition to revision ACL reconstruction in their failure cohort [25]. There were 81 reported patient failures among the 614 included patients with a minimum two-year follow-up (failure rate, 13.2%). The failure rates for the SA, NA, and DIS techniques were 12.6% (33/261), 9.4% (17/179), and 17.8% (31/174) (p = 0.090), respectively (Table 3). Based upon a chi-squared analysis, these values were not significantly different.

**Table 3:** Primary ACL repair failures with IKDC and Lysholm scores at the final follow-up Jonkergouw et al. [29] and Vermeijden et al. [36] are listed twice as they both compared SA versus NA. *N/A: information was not included in the study. ACL: anterior cruciate ligament, IKDC: International Knee Documentation Committee

Author (year)	Type of Primary Repair	Number of Failures n=	Failure Rate (%)	IKDC (range)	Lysholm (range)
Achtnich et al. 2016 [19]	Non-Augmented	3	14.30%	N/A	N/A
Bigoni et al. (2017) [21]	Non-Augmented	0	0%	93.3 (67.8-95)	93.6 (68-100)
Jonkergouw et al. 2019 [29]	Non-Augmented	4	13.8%	90.6	95.2
Liao et al. 2020 [32]	Non-Augmented	1	4.7%	86.0 (57-100)	93.6 (80-100)
Liao et al. 2020 [33]	Non-Augmented	0	0%	88.3 (79-100)	93.1 (86-100)
Mukhopadhyay et al. 2018 [34]	Non-Augmented	0	0%	N/A	95.0
Turati et al. 2021 [35]	Non-Augmented	4	21.1%	95.7	93.5
Vermeijden et al. 2021 [36]	Non-Augmented	5	9.4%	N/A	94.6
Burton et al. 2021 [22]	Suture Augmented	2	7.14%	N/A	N/A
Dabis et al. 2020 [23]	Suture Augmented	0	0%	N/A	95.0 (90-100)
Douoguih et al. 2020 [24]	Suture Augmented	4	14.80%	N/A	N/A
Gagliardi et al. 2019 [25]	Suture Augmented	9	48.80%	90.8	96.2
Heusdens et al. 2019 [26]	Suture Augmented	2	4.80%	N/A	N/A
Hopper et al. 2022 [27]	Suture Augmented	6	17.60%	N/A	N/A
Jonkergouw et al. 2019 [29]	Suture Augmented	2	7.4%	89.4	93.0
Vermeijden et al. 2021 [36]	Suture Augmented	8	13.3%	N/A	94.6
Ahmad et al. 2020 [20]	Dynamic Stabilization	17	30%	94.0 (63.2-100)	94 (64-100)
Hoogeslag et al. 2019 [28]	Dynamic Stabilization	2	8.7%	95.4	N/A
Kohl et al. 2016 [30]	Dynamic Stabilization	5	10%	97.6	99.3
Kösters et al. 2020 [31]	Dynamic Stabilization	7	16.3%	90.0	92.0

There were 13 studies that reported their technique to assess ATT postoperatively. There were eight studies that utilized arthrometers (n = 7, KT-1000 [19-21,25,30,34,35]; n = 1, Rolimeter (aircast) testing [31]). Seven of these studies reported specific measurements of ATT, which ranged from 0.8 mm [20] to 3.0 [21]. Three studies utilized physical exams, such as Lachman's maneuver test or anterior drawer test to assess ATT [23,32,33]. There were three studies that used the NA technique, which reported ATT using an arthrometer and reported 1.95 [19] and 3 mm [21] of ATT. There were three studies that reported ATT after the DIS and reported a range of 0.8 [20]-1.9 [31] (this study used a rolimeter) mm of ATT. Finally, there was one study that reported a mean ATT of 1.2 mm using Lachman’s test [23].

Comparative Studies in ACL Reconstruction

There were four studies that compared primary repair to reconstruction [19,25,28,31]. Three of these studies found no significant differences in failure rate between repair and reconstruction [19,28,31] (p-values = 0.663, 0.432, 0.321). Gagliardi et al. [25] analyzed primary ACL (22 patients) versus ACL reconstruction (157) in pediatric patients and found a failure rate of 48.8-4.8% (p = 0.0001). Kösters et al. [31] found a significantly greater ATT in DIS versus ACL reconstruction (1.9 mm vs. 0.9 mm; P = 0.0086), but there was no significant difference in PROs. Among these four studies, there was no statistical difference in PROs between primary repair and ACL reconstruction [19,25,28,31].

Results of Meta-Analysis

**Figure 2:**
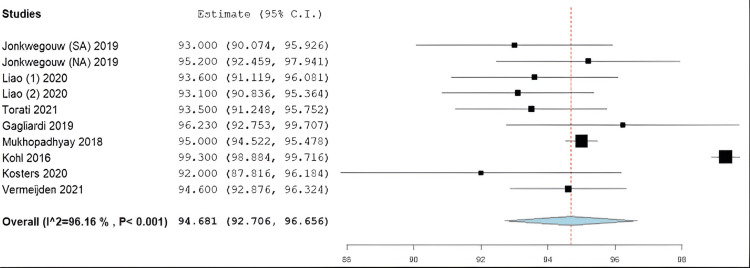
Forest plot for the Lysholm scores of patients following ACL repair SA: suture augmentation, NA: no suture augmentation Jonkergouw et al. (2019) [29], Liao and Zhang (2020) [32], Liao and Zhang (2020) [33], Turati et al. (2021) [35], Gagliardi et al. (2019) [25], Mukhopadhyay et al. (2018) [34], Kohl et al. (2016) [30], Kosters et al. (2020) [31], Vermeijden et al. (2021) [36]

**Figure 3:**
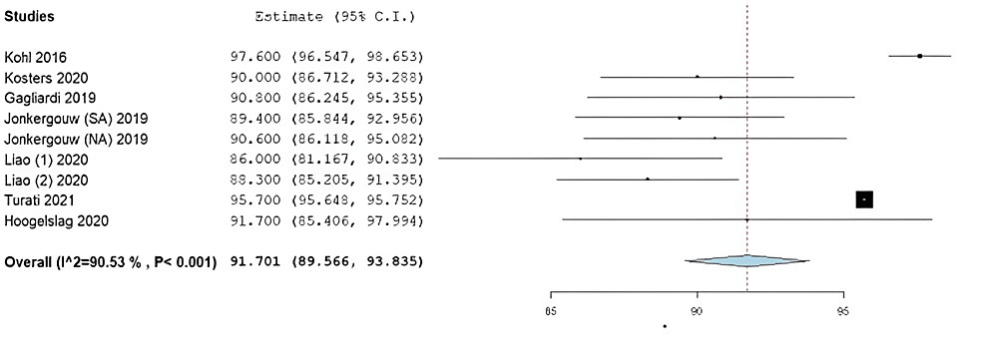
Forest plot for the International Knee Documentation Committee (IKDC) scores SA: suture augmentation, NA: no suture augmentation Kohl et al. (2016) [30], Kosters et al. (2020) [31], Gagliardi et al. (2019) [25], Jonkergouw et al. (2019) [29], Liao and Zhang (2020) [32], Liao and Zhang (2020) [33], Turati et al. (2021) [35], Hoogeslag et al. (2020) [28]

**Figure 4:**
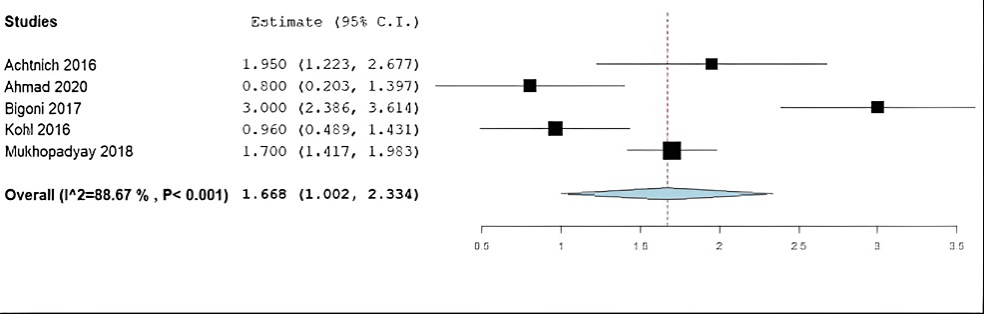
Forest plot for the amount of anterior tibial translation (ATT) at the latest available follow-up following anterior cruciate ligament repair All measurements were recorded using a KT-1000 (knee arthrometer), and the measurements were taken in millimeters. Achtnich et al. (2016) [19], Ahmad et al. (2020) [20], Bigoni et al. (2017) [21], Kohl et al. (2016) [30], Mukhopadyay et al. (2018) [34]

The average postoperative Lysholm score among the included studies was 94.7 (95% CI: 92.7-96.7) (Figure 2). The average IKDC score was 91.7 (95% CI: 89.6-93.8) (Figure 3). Finally, the average ATT among the entire study population was found to be 1.67 mm (95% CI: 1.0-2.3 mm) (Figure 4). It is important to note that only those studies reported ATT measurements obtained using the KT-1000 arthrometer.

Discussion

This study was successful in achieving its primary purpose, which was to evaluate the clinical outcomes and failure rates of primary repair for proximal ACL tears. We found no statistically significant difference between failure rates or PRO scores among the different types of primary ACL repair techniques. This analysis shows that primary ACL repair has encouraging short-midterm outcomes for patients with proximal ACL injuries. The review also found that repair in the pediatric population is associated with a higher failure rate. As such, this study shows that primary ACL repair is a reasonable option in managing proximal ACL ruptures, and further randomized controlled clinical trials are warranted.

Failure Rates

We found a combined failure rate of 12.6% among patients who underwent primary ACL repair of proximal ACL ruptures with a minimum two-year follow-up. When grouping just the SA and NA techniques together, the failure rate drops to 11.4%, although there is no statistical difference between the different groups. This failure rate is comparable to other recent systematic reviews, which report failure rates ranging from 7% to 11% at a mean follow-up of two years among the different techniques [40,41]. One of these studies done by Vermeijden et al. [41] analyzed SA primary repair (8% failure rate), while the other by Van der List et al. [40] compared DIS versus NA versus SA repair (11%, 10%, and 7% failure rate) and found no statistical difference between the types of primary repair. While the current authors admit that there is a high risk of bias and no randomized controlled clinical trials included [42], the current analysis found that primary repair has satisfactory outcomes at short-midterm follow-up. The findings of the current study justify the need for future randomized controlled trials designed to examine the outcomes of primary ACL repair and compare the different repair techniques.

Primary ACL Repair Versus Reconstruction

Among the studies included in the current systematic review, four compared primary ACL repair to reconstruction [19,25,28,31]. Of 351 patients included in the four studies, only 31% underwent primary ACL repair. Of these four studies, only Kösters et al. [31] reported a statistically significant difference in ATT at 0.9 mm in ACL reconstruction and 1.9 mm among those patients who underwent primary repair with the DIS technique. Our study showed a combined ATT of 1.7 mm, which is similar to a reported 1.7 mm ATT of a study on ACL reconstruction [43]. Among the four studies that compared ACL repair and reconstruction, no significant differences were found when analyzing PROs, such as the IKDC, Lysholm, or Tegner, between ACL reconstruction and primary repair [19,25,28,31]. In regard to failure rates, only one study showed a significant difference with 48.8% failing in the primary repair group and only 4.8% in the ACL reconstruction [25]. Of note, Gagliardi et al.'s study [25] was exclusively on pediatric patients and only included 22 patients undergoing primary repair. Among these studies, only one showed a significant difference between failure rates and ATT, with no statistical differences identified in any of the four studies with respect to PROs. Moreover, the literature suggests that ACL repair offers a favorable profile compared to ACL reconstruction including, the fact that it is less invasive, it is associated with less morbidity, and the theoretically improved proprioceptive potential [14,15].

Primary ACL Repair in Pediatric Patients

In response to the high failure rates among the pediatric population in Gagliardi et al.'s [25] study, Dabis et al. [23] proposed that the abnormally elevated failure rates resulted from the surgical technique used by Gagliardi et al. [25]. They claimed that with the use of multiple 4 mm femoral and tibial drill tunnels, it is likely that the ACL or ACL footprint would have been damaged. Dabis et al. [23] examined 20 pediatric patients (ages 6-16) and found no differences in failure rate or complications at the two-year follow-up. Although the surgical technique used in Gagliardi et al.'s [25] study could have played a role in the significantly elevated failure rate, other recent literature has demonstrated an increased risk of failure among pediatric patients undergoing primary ACL repair [27,36,44]. Another study done by Hopper et al. [27] found that 17.6% of patients failed by five years with a significant difference in failures occurring in the younger patient population. The authors suggested that this difference was because younger patients are more active. The activity was identified by significant differences in pre-injury Marx activity scores. Similarly, Vermeijden et al. [36] reported an overall failure rate of 11.5% with a significantly larger portion of failures observed in patients <21 years old. It appears that pediatric patients undergoing primary ACL repair are at an increased risk of failure.

PROs of Primary ACL Repair

The current study identified encouraging PRO metrics among the included patients. The average Lysholm score of 94.60 in this study is similar to that found in the literature regarding normal knees. While “normal” is subjective, previous studies have reported this number to be around 95 [45,46]. In addition, the average IKDC score in knees that returns to perceived normal function after ACL surgery is likely between 91 and 95 [47]. Our study found an average IKDC score of 91.7 at the final follow-up, which falls within that normal range. Furthermore, the average IKDC score of this study falls above what has been determined to be an acceptable symptom state (85.1±10) [48], supporting the use of ACL repair for proximal ACL ruptures in indicated patients

Limitations

We acknowledge that limitations are present in this systematic review. Although primary ACL repair has regained momentum in the last five to 10 years, we were only able to include 18 studies that met our inclusion/exclusion criteria with only 614 total patients. A large variety of heterogeneity was present among the studies included with patients ranging from six to 65 years old and failure rates being defined differently among the different studies. Furthermore, of the 18 studies, only two were level I randomized controlled clinical trials with the others being mainly smaller non-comparative case-control studies. Despite our attempts to homogenize included studies, we acknowledge that there is a high amount of bias among the included studies in this meta-analysis. Although our systematic review required a longer follow-up than previous systematic reviews on primary repair, a limitation present is the lack of mid-long-term follow-up results for primary ACL repair. Studies have shown failures are more likely to happen within a few years of ACL repair and the incidence of failure will often flatten out around five years, which is what we recommend for future studies [20,49].

## Conclusions

The primary findings of this paper include a 12.6% combined failure rate for primary repair with no significant difference in failure rate or PROs when accounting for the methodology of repair at a minimum two-year follow-up. It is important to note the lack of high-quality randomized controlled trials, the heterogeneity of included studies, and the lack of long-term data. Despite these limitations, the findings of the current analysis suggest that primary repair may be a useful treatment option for indicated candidates with proximal ACL ruptures. Further long-term and comparative studies to ACL reconstruction are warranted.
